# Activity engagement and loneliness among older adults who live alone: Heterogeneity by race/ethnicity

**DOI:** 10.1093/geroni/igaf122.3107

**Published:** 2025-12-31

**Authors:** Maki Karakida, Sung Park, Sara Minemoto, Richard Viskochil

**Affiliations:** University of Massachusetts Boston, Boston, Massachusetts, United States; University of Massachusetts Boston, Boston, Massachusetts, United States; University of Massachusetts Boston, Boston, Massachusetts, United States; University of Massachusetts Boston, Boston, Massachusetts, United States

## Abstract

Social and physical activities play a key role in reducing a feeling of loneliness among older individuals, especially those living alone. Yet, little is known about the associations between activity engagement and loneliness symptoms in older U.S. adults living independently by race/ethnicity. This longitudinal study examines the relationships between activity engagement (social, household, and physical activity; volunteering) in relation to race/ethnicity and loneliness among community-dwelling Americans aged 70+ who live alone. Using the most recent data from the National Health and Aging Trends Study (NHATS), we examined associations between activity engagement and levels and loneliness by race/ethnicity, accounting for sociodemographic, health and other characteristics. Results highlighted that the Hispanic group consistently showed significantly higher loneliness symptoms than their non-Hispanic White peers, even after adjusting for covariates. On the contrary, the non-Hispanic Black group reported significantly lower loneliness scores than non-Hispanic White persons. Activity engagement also signaled a significantly lower likelihood of loneliness across all the models. The model examining activity engagement and race/ethnicity together demonstrated that, compared to the non-Hispanic White group, non-Hispanic Black adults had significantly higher rates of loneliness whereas Hispanic adults indicated significantly lower levels of feeling lonely. These findings suggest that among older U.S. adults living alone, race/ethnicity in conjunction with social and physical activity involvement may lead to differential impacts on their loneliness outcomes and support the development of race/ethnicity-specific interventions.

